# Retraction: Somatostatin Derivative (smsDX) Targets Cellular Metabolism in Prostate Cancer Cells after Androgen Deprivation Therapy

**DOI:** 10.1371/journal.pone.0299822

**Published:** 2024-02-26

**Authors:** The PLOS ONE Editors

Following the publication of this article [1], concerns were raised regarding results presented in Fig 2. Specifically,

- Fig 2A: the LNCaP Vehicle 0h panel appears to partially overlap with the smsDX(1nM) 0h panel.
- Fig 2B: the LNCaP-S Vehicle 0h panel appears to partially overlap with the smsDX(1nM) 0h panel.

Following editorial communication, the corresponding author Zhaoxu Liu stated that the original data underlying the article are no longer available and requested retraction of the article.

In light of the above concerns, the *PLOS ONE* Editors retract this article.

Following the discussion of these concerns, Anders Holmberg and Sten Nilsson contacted PLOS stating that they were unaware they were listed as coauthors on the article; that the study was not carried out in affiliation with the Karolinska Institutet; and that a non-existent email address had been provided with the submission for Anders Holmberg. Corresponding author Zhaoxu Liu confirmed that Anders Holmberg and Sten Nilsson were added to the manuscript without their knowledge or consent, and that the study did not receive any support from the Karolinska Institutet.

The Karolinska Institutet confirmed that Anders Holmberg, Sten Nilsson, and the Karolinska Institutet were not, at any point, involved in the study. In light of the institute’s findings, this article was republished at the time of Retraction to remove Anders Holmberg and Sten Nilsson from the author list, and to remove the Karolinska Institutet from the affiliation list of author Zhaoxu Liu.

The authors did not respond to express their agreement or disagreement with the retraction decision.
